# Residents learning ultrasound-guided catheterization are not sufficiently skilled to use landmarks

**DOI:** 10.1186/cc13741

**Published:** 2014-02-23

**Authors:** Julien Maizel, Laurianne Guyomarc’h, Pierre Henon, Santhi Samy Modeliar, Bertrand de Cagny, Gabriel Choukroun, Michel Slama

**Affiliations:** 1 Medical Intensive Care Unit, Department of Nephrology, Amiens University Medical Center, Amiens, France; 2 INSERM U-1088, Jules Verne University of Picardie, Amiens, France

## Abstract

**Introduction:**

Ultrasound-guided (UG) technique is the recommended procedure for central venous catheterization (CVC). However, as ultrasound may not be available in emergency situations, guidelines also propose that physicians remain skilled in landmark (LM) placement. We conducted this prospective observational study to determine the learning curve of the LM technique in residents only learning the UG technique.

**Methods:**

During the first three months of their rotation in our ICU, residents inexperienced in CVC used only the real-time UG technique. During the following three months, residents were allowed to place CVC by means of the LM technique when authorized by the attending physician.

**Results:**

A total of 172 procedures (84 UG and 88 LM) were performed by the inexperienced residents during the study. The success rate was lower (72% versus 84%; *P* = 0.05) and the complication rate was higher (22% versus 10%; *P* = 0.04) for LM compared to UG procedures. Comparison between the five last UG procedures and the first five LM procedures performed demonstrated that the transition between the two techniques was associated with a marked decrease of the success rate (65% versus 93%; *P* = 0.01) and an increase of the complication rate (33% versus 8%; *P* = 0.01). After 10 LM procedures, residents achieved a success rate and a complication rate of 81% and 6%, respectively.

**Conclusions:**

Residents who only learn the UG technique will not be immediately able to perform the LM technique, but require specific training based on at least 10 LM procedures. The question of whether or not the LM technique should still be taught when an ultrasound device is not available must therefore be addressed.

## Introduction

In teaching hospitals in many countries, central vein cannulation (central venous catheters (CVC) or dialysis catheters) is usually performed by residents. Whenever possible, the real-time ultrasound-guided (UG) technique has become the recommended procedure for central vein catheterization in ICU and emergencies, because it increases the success rate, and decreases the complication rate, the procedure time and the cost [1-5]. According to guidelines, novice residents should start by learning the UG technique. However, the American Society of Echocardiography, the Society of Cardiovascular Anesthesiologists, the National Institute for Clinical Excellence and the American Society of Anesthesiologists all recognize that, in certain circumstances, such as emergency situations, the use of ultrasound may be impossible (because unavailable) and that operators must therefore maintain their skills by placing central catheters according to the landmark (LM) technique for these specific situations [1-3]. Some authors have suggested that the anatomical knowledge gained by using the ultrasound technique improves the operators’ skills when they need to use the LM technique in an emergency [6]. However, this opinion is based exclusively on personal experience and not on any clinical data. It could also be harmful for physicians who have only learned the UG technique to consider themselves sufficiently skilled to attempt an LM procedure in an emergency situation. In our experience, residents performing only UG catheterization are not able to achieve the LM technique and therefore require additional training. In our ICU, inexperienced residents exclusively learn the UG technique during the first 3 months of their rotation and are then allowed to learn the LM technique during the following 3 months under the supervision of the attending physician. The objective of this study was to determine the learning curve of the LM technique in residents who have only been trained in the UG technique.

## Methods

In accordance with French legislation, the local institutional review board (CPP Nord-Ouest II, Amiens University Hospital, France) approved the study protocol and considered no written informed consent was required, as teaching the LM technique is recommended and part of our regular practice. Information was given and oral consent obtained from patients or their relatives about the purpose of this prospective observational study and the anonymous use of the parameter recorded for scientific publication. Over a 3-year period (May 2008 to April 2011), all residents with no experience of catheter placement (fewer than three attempts) working in our medical ICU (Amiens University Hospital, France) were included in this study. All residents included in the study were followed during their 6-month rotation in our unit. During the first 3 months, the residents were only allowed to use the UG technique. During the following 3 months, residents were allowed to perform CVC placement by the LM technique under the supervision of the attending physician. The decision to allow the resident to use the LM procedure was based on the risk of complications and the patient’s condition, as assessed by the attending physician. All CVC or dialysis catheters, femoral or jugular procedures performed by these residents were prospectively recorded during this 6-month period (except for UG procedures performed during the LM period).

Subclavian catheter placement is rarely performed in our department and never by trainee residents because of the high risk of pleural puncture, and because many patients in our ICU (part of the nephrology department) require careful preservation of their capital vein in case a fistula is subsequently required. The central catheter placement-learning program in our department was established according to guidelines [1]. Before starting their first procedure, all residents received theoretical training on ultrasound, the ultrasound apparatus and central line placement using the UG technique. For that purpose they also watched the video provided by the *New England Journal of Medicine* named ‘Placement of a femoral venous catheterʼ, which is available online. Because we do not have any inanimate models, the residents learned on patients how to manipulate the probe to correctly visualize the different vessels, and then performed a four-hands procedure with the attending physician. After this educational training program (including the four-hands procedure), they were allowed to perform their first procedure. At the beginning of the fourth month, when residents started to perform LM procedures, they received an additional tutorial on the anterior LM technique. All UG and LM procedures were performed under the direct supervision of a senior physician.

### Real-time ultrasound-guided procedure

An ultrasound device designed for ultrasound-guided puncture was used (Site-Rite 5, Dymax Corp., Salt Lake City, Utah, USA). Prior to cannulation, various sites (right and left jugular and femoral veins) were rapidly examined to determine the optimal approach for catheter placement. The skin was cleaned with povidone-iodine (Betadine) with alcohol before placement of sterile drapes. The operators wore a gown, cap, mask and sterile gloves.

A 7.5-MHz linear array probe was covered by a sterile sheath and was connected to a two-dimensional image display. The real-time ultrasound technique has been extensively described elsewhere [7]. Briefly, transverse ultrasound imaging allows identification of the carotid and femoral arteries and internal jugular and femoral veins by their relative position, compressibility, and expansion during inspiration and visible pulsation of the artery. After anesthetizing the skin (1% lidocaine), a 19-gauge, 6.35-cm-long needle connected to a 10-ml syringe was advanced through the skin, using a needle guide attached to the transducer, as described elsewhere, under real-time ultrasound guidance [7].

### Landmark procedure

The operator and attending physician decided on the site of placement without ultrasound guidance. The skin was cleaned with povidone-iodine (Betadine) with alcohol before placement of sterile drapes. The operators wore a gown, cap, mask and sterile gloves. 1% lidocaine infiltration was performed at the puncture site before starting the procedure. The right (or left) internal jugular or femoral vein was punctured with a 19-gauge, 6.35-cm-long needle connected to a 10-ml syringe. For jugular cannulation, subjects were placed in the supine position with the head rotated 30°. The usual puncture site was located in the neck, 4 cm below the angle of the mandible at the level of the thyroid cartilage and at the medial border of the sternocleidomastoid muscle, lateral to the common carotid artery. The needle was then inserted under the sternocleidomastoid muscle, aiming for the junction of the middle and medial thirds of the clavicle, with a 45° posterior angle of entry with the skin. For femoral access, the external LM-guided technique was performed by manual localization of the femoral artery in the femoral triangle inferior to the inguinal ligament with needle insertion medial to the artery.

### Data collection

Patient characteristics (body mass index (BMI), blood pressure, heart rate), type of catheter (CVC or dialysis catheter), site of catheterization (femoral or jugular), simplified acute physiologic score 2 (SAPS2), prothrombin time, activated partial thromboplastin time and platelet count were recorded. A puncture attempt was defined as a separate skin puncture. Successful placement was defined when the catheter was fully inserted within a maximum of three punctures. The venous return time was the time between the first penetration of the skin and aspiration of venous blood into the syringe, allowing insertion of the guide wire. The procedure time was the interval between first penetration of the skin and complete insertion of the device into the vein (before connecting the lines and before fixation). Mechanical complications included hematoma (visible or palpable modification of the skin relief by a blood collection) and arterial puncture (aspiration of pulsatile arterial blood).

### Statistical analysis

Results are expressed as mean ± SD or number (proportions), as appropriate. As the same residents performed both the UG and LM procedures, the two groups cannot be considered to be independent. Moreover, all residents did not perform the same number of UG and LM procedures. Proportions and means were therefore compared by using a generalized estimating equations procedure (GEE). GEE is an extension of the generalized linear model, which allows analysis of repeated measurements (in this case, the procedures performed by the same resident). A moving-average method was used to analyze learning curves. A moving average of 3 was used to attenuate variations and accentuate trends. Statistical analysis was performed using IBM SPSS (version 15, IBM, USA). The limit of significance was set at *P* ≤0.05.

## Results

### Patient characteristics

The characteristics of the 172 procedures (84 UG and 88 LM) recorded during the study are presented in Table 1. No significant difference in SAPS2, BMI, heart rate, blood pressure, prothrombin time, activated partial thromboplastin time, platelet count and proportion of CVC/dialysis catheters was observed between the 2 groups. A higher proportion of femoral procedures was observed in the LM group compared to the UG group (52 (59%) vs 35 (42%), p = 0.05, respectively).

**Table 1:** Baseline characteristics

**Parameters**	**Ultrasound-guided n = 84**	**Landmark-guided n = 88**	***P*-value**
SAPS2	55 ± 18	55 ± 20	0.8
Body mass index, kg/m^2^	30 ± 10	28 ± 6	0.2
Heart rate, bpm	92 ± 20	98 ± 23	0.07
SBP, mmHg	113 ± 30	116 ± 30	0.3
DBP, mmHg	60 ± 17	59 ± 17	0.6
Prothrombin time, %	63 ± 21	64 ± 19	0.1
Activated partial thromboplastin time, sec	33 ± 8	36 ± 17	0.2
Platelet count, 10^3^/mm^3^	186 ± 120	188 ± 122	0.9
Type of catheter (central venous catheter / dialysis catheter), n	49 (58)/35 (42)	41 (47)/47 (53)	0.3
Site (jugular/femoral), n	49 (58)/35 (42)	36 (41)/52 (59)	0.05

Results are presented as mean ± SD, or number (n). SAPS2, Simplified Acute Physiologic Score 2; SBP, systolic blood pressure; DBP, diastolic blood pressure.

### Operators and procedures

Eight residents, all in their fourth or fifth year of medical residency, were included in this study. Each resident performed an average of 11 ± 2 procedures according to the UG technique during the first 3 months and 11 ± 2 procedures according to the LM technique during the last 3 months. They also performed 2 ± 2 additional UG procedures during the last 3 months (either because the attending physician contraindicated the LM technique or as a rescue technique after failure of the LM technique), but these procedures were not included in the statistical analysis. Comparisons of outcome measures between the two techniques are shown in Table 2. LM procedures were associated with a lower success rate (72% versus 84%; *P* = 0.01), a higher complication rate (22% versus 10%; *P* = 0.01) and a higher mean number of attempts (1.8 ± 0.9 versus 1.6 ± 0.8; *P* = 0.001) compared to the UG technique, but venous return time was similar (1.7 ± 3.2 versus 1.4 ± 2.5 minutes; *P* = 0.5) between the two groups. The procedure time was significantly shorter in the LM technique compared to the UG technique (5.1 ± 3.4 versus 6.7 ± 5.0 minutes; *P* = 0.02). The success rate (72% versus 71%; *P* = 0.9, respectively) and complication rate (22% versus 21%; *P* = 0.9, respectively) between the jugular and femoral sites in the LM group were similar. The higher proportion of femoral procedures in the LM group (Table 1), therefore, did not constitute a drawback for this technique.

**Table 2:** Comparison of outcome measures in the ultrasound-guided and landmark procedures

	**Ultrasound-guided n = 84**	**Landmark n = 88**	***P*-value**
Success rate, n (%)			
All	71 (84)	63 (72)	0.01
Jugular	40 (82)	26 (72)	0.1
Femoral	31 (82)	37 (71)	0.01
Complication rate (hematoma and arterial puncture), n (%)			
All	8 (10)	19 (22)	0.01
Jugular	5 (10)	8 (22)	0.04
Femoral	3 (9)	11 (21)	0.07
Arterial puncture, n (%)			
All	6 (7)	16 (18)	0.05
Jugular	4 (8)	6 (17)	0.4
Femoral	2 (6)	10 (19)	0.1
Procedure time, minutes, mean ± SD (n = 71/63)	6.7 ± 5.0	5.1 ± 3.4	0.02
Venous return time, minutes, mean ± SD (n = 74/71)	1.4 ± 2.5	1.7 ± 3.2	0.5
Number of attempts, mean ± SD	1.6 ± 0.8	1.8 ± 0.9	0.001

To analyze the transition between UG and LM procedures in more detail, we compared the outcomes of the last five UG procedures and the first five LM procedures performed by the residents (Table 3). Individual results for each resident are also shown in Table 3. The global success rate at the end of the UG period was 93% and the complication rate was 8%. The success rate was significantly lower (65% versus 93%; p = 0.01) and the complication rate was significantly higher (33% versus 8%; p = 0.01) for the first 5 LM procedures. The venous return time and the number of attempts were also increased during the first 5 LM procedures compared to the last 5 UG procedures (2.7 ± 4.4 versus 1.0 ± 1.8 minutes; *P* = 0.05 and 2.0 ± 0.9 versus 1.5 ± 0.7; *P* = 0.01, respectively) although the procedure times were similar (5.9 ± 3.8 versus 6.1 ± 3.8; *P* = 0.8).

**Table 3:** Outcome measures in the last five ultrasound-guided procedures and the first five landmark procedures

		**Last 5 ultrasound-guided (UG) procedures**					**First 5 landmark (LM) procedures**				
**Resident**	**Number of procedures (UG/LM)**	**Success rate**	**Complication rate**	**Venous return time, minutes**	**Procedure time, minutes**	**Number of attempts**	**Success rate**	**Complication rate**	**Venous return time, minutes**	**Procedure time, minutes**	**Number of attempts**
1	10/9	80%	20%	1.3 ± 2.5	6.5 ± 3.5	1.6 ± 0.9	40%	0%	1.6 ± 0.8	7.7 ± 2.5	2.0 ± 1.0
2	11/9	60%	0%	0.3 ± 0.5	1.8 ± 0.3	1.8 ± 1.1	60%	60%	6.0 ± 7.9	8.7 ± 8.1	2.4 ± 0.9
3	8/9	100%	20%	3.0 ± 2.8	6.4 ± 3.8	1.6 ± 0.5	40%	40%	0.1 ± 0.1	7.5 ± 0.7	1.6 ± 0.9
4	9/11	100%	0%	0.5 ± 0.6	8.1 ± 3.3	1.2 ± 0.4	60%	40%	2.6 ± 3.7	3.5 ± 0.9	2.0 ± 1.0
5	9/9	100%	0%	0.5 ± 0.2	4.3 ± 1.4	1.0 ± 0.1	80%	20%	1.7 ± 2.2	3.1 ± 2.6	1.4 ± 0.5
6	14/11	100%	0%	0.2 ± 0.2	8.4 ± 5.0	1.6 ± 0.9	80%	20%	1.0 ± 2.0	5.6 ± 0.9	2.0 ± 1.0
7	12/13	100%	0%	1.1 ± 1.7	3.9 ± 2.3	1.4 ± 0.9	100%	0%	0.6 ± 0.3	4.0 ± 1.7	1.8 ± 0.8
8	11/15	100%	20%	1.2 ± 2.4	8.0 ± 4.6	1.4 ± 0.5	60%	80%	8.3 ± 7.5	10.0 ± 5.0	2.4 ± 0.9
All	11 ± 2/11 ± 2	93%	8%	1.0 ± 1.8	6.1 ± 3 .8	1.5 ± 0.7	65%^a^	33%^a^	2.7 ± 4.4^a^	5.9 ± 3.8	2.0 ± 0.9^a^

Venous return time, procedure time and number of attempts are presented as mean ± SD. ^a^*P* ≤0.05 versus last 5 UG procedures.

**Figure 1:**
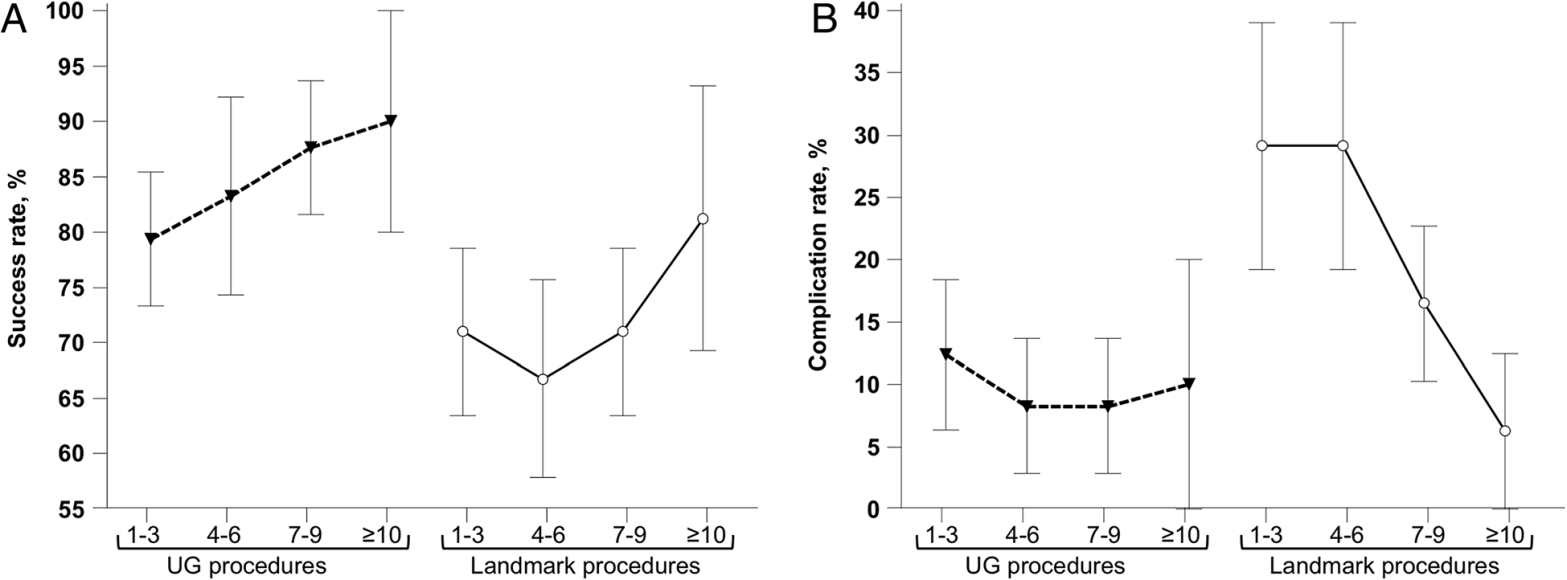
**Time course of the success rate and complication rate according to the number of procedures performed by each resident. (A)** Success rate; **(B)** complication rate. Results are presented as mean and standard error of the mean (SEM).

The course of success rates and complication rates according to the number of procedures performed is represented in Figure 1. These curves represent the UG technique learning curve followed by a dramatic decrease in the success rate and an increase in the complication rate when residents performed their first LM procedures. It also illustrates that residents subsequently improved their catheterization skills when using the LM technique, achieving a success rate of 81% and a complication rate of 6% after 10 procedures.

**Figure 2:**
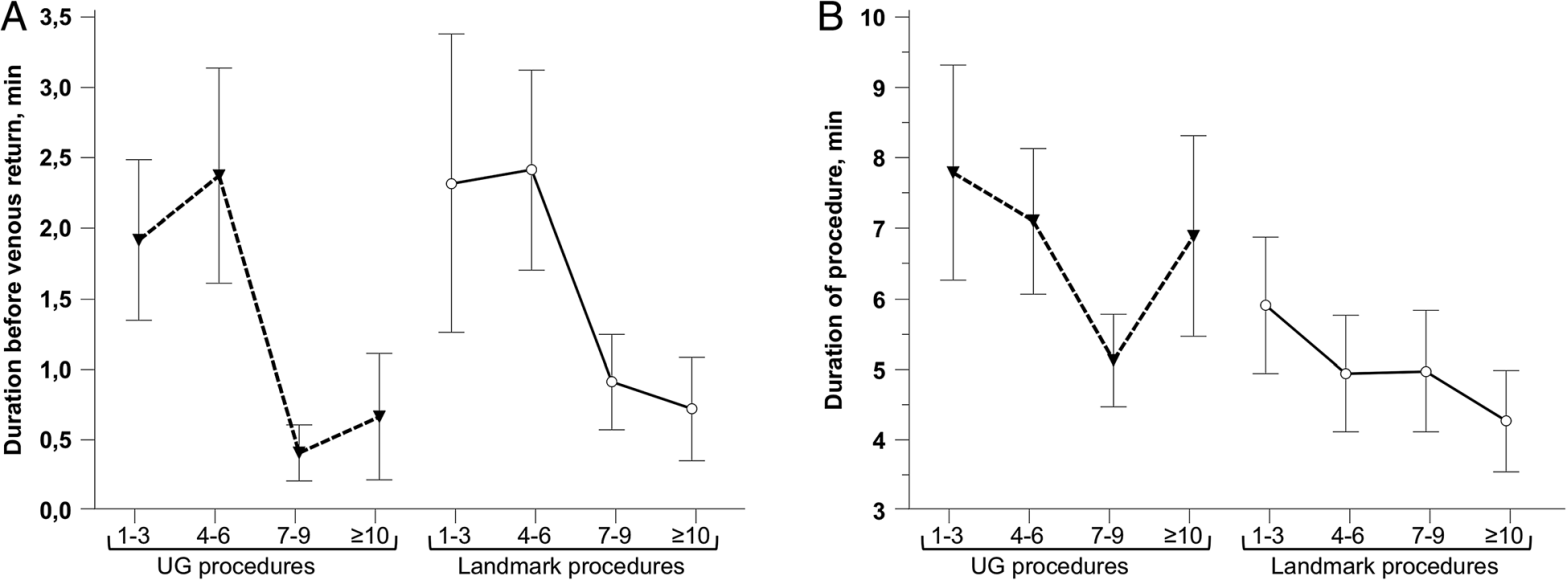
**Time course of venous return time and procedure time according to the number of procedures performed by each resident. (A)** Venous return time; **(B)** procedure time. Results are presented as mean and standard error of the mean (SEM).

Interestingly, the venous return time decreased progressively with increasing experience with the UG technique followed by a dramatic increase when residents started to perform the LM technique (Figure 2). Venous return time subsequently improved with the number of LM procedures performed. Inversely, procedure time did not increase during the first LM procedures and continued to decrease at the same rate as during the first 3 months of the exclusive UG technique (Figure 2). Transition from the UG technique to the LM technique, therefore, altered the residents’ capacity to find the central vein, but not their capacity to place the device in the vein once the vein had been found.

## Discussion

The UG technique has been shown to improve the safety and efficacy of CVC placement in ICU patients compared to the LM technique and is now the most broadly recommended procedure [1-5]. However, in some emergency situations, an ultrasound device may not be available. Physicians must therefore be able to perform CVC placement according to the LM technique. It is unclear whether residents who have only learned the UG technique are also able to perform the LM technique. The present study clearly demonstrates that residents who had only learned the UG technique were not immediately able to perform the LM technique, but required specific training in this technique, comprising at least 10 LM procedures.

Previous studies by our team and other authors have already demonstrated the benefits of the UG technique for physicians inexperienced in CVC placement, especially during the first procedures when UG can markedly decrease the complication rate [8-10]. The UG learning curve is steeper than the LM learning curve. In other words, residents achieve success earlier with the UG technique with fewer complications. Therefore, by exclusively teaching the UG technique, patients would be managed by the safer of the two procedures. However, such an approach means that young physicians may be unable to perform catheter placement without ultrasound guidance, especially in emergency situations.

This study, confined to novice residents, addressed this issue and clearly showed that training exclusively in the UG technique does not provide sufficient skills to perform the LM technique. This study also showed that the residents’ skills rapidly improve after they have performed 10 procedures with a success rate (81%) and complication rate (6%) comparable to those observed in previous studies of physicians learning the LM technique [7,10].

These results also demonstrate that learning the UG technique provides residents with certain skills to perform LM catheterization, as first the residents only needed to perform 10 LM procedures to achieve satisfactory success and complication rates and second the procedure time was not increased when residents used the LM technique although venous return time increased. Use of UG helps the physician to puncture the central vein. The venous return time is therefore prolonged when switching to the LM technique, but once the needle is in the vein, the catheter insertion is the same using the UG technique and the LM technique. Figure 2 also shows that the procedure time, which includes venous return time and catheter insertion, improved continuously with the number of procedures, even when the resident switched to the LM technique, demonstrating that the resident acquires certain skills during UG procedures that are useful for central catheter insertion.

This acquisition of skills also explains why a shorter procedure time was observed in the LM group. In our study, UG procedures were performed before LM procedures, so residents had already acquired a certain amount of experience when they started to perform LM procedures. In previously published studies that demonstrated a gain of time with UG compared to LM, the two techniques were randomly assigned avoiding any difference of experience between groups [3,8].

This study shows the need to teach the LM technique to residents already skilled in the UG technique so that they will be able to perform catheter placement when an ultrasound machine is not immediately available. However, our study also demonstrates that, even after the resident has acquired good skills with the UG technique, the learning phase of the LM technique is still associated with significant complication and failure rates. The results of this study therefore raise a more general issue. Instead of teaching residents to use the LM technique when ultrasound is not available, which exposes patients to a risk of complications, alternative emergency approaches, such as intra-osseous lines could be considered [11]. The use of simulators, which have already been shown to improve physician training in cannulation techniques, could also be a safer alternative to teach the LM technique [12,13]. Future investigations should address these questions raised by our study.

We did not address the issue of maintenance of these LM procedure skills, notably whether experienced physicians should occasionally perform the LM technique in order to maintain their skill. This study concerned various sites (femoral and jugular) and types of catheterization (CVC or dialysis catheter). The study could not have been designed otherwise, as residents are trained simultaneously in jugular and femoral vein catheterization and the learning curve depends on each procedure regardless of the site. During the LM technique period, the attending physician was able to oblige the resident to use the UG technique. This decision was based on evaluation of the risk factors and may have constituted a selection bias in favor of the LM group, as patients in the LM group would have presented fewer risk factors for complications. However, the two groups presented similar risk factors (Table 1). The only difference between the two groups was a higher proportion of femoral procedures in the LM group. Success rates (72% versus 71%, respectively; *P* = 0.9) and complication rates (22% versus 21%; *P* = 0.9, respectively) were similar between jugular and femoral procedures in the LM group (Table 2). We therefore consider that this selection did not constitute a significant bias in our study. Our LM procedure uses the thyroid cartilage as the palpation landmark, whereas other teams recommend using the cricoid cartilage. To the best of our knowledge, no data are available in the literature in favor of the use of one or other of these cartilages and we consider the thyroid cartilage to be more easily palpable. Our results in the UG procedure were obtained using an ultrasound machine with a needle guide attached to the transducer, a device specifically dedicated for the placement of CVC [7].

## Conclusion

The UG technique is the first technique that should be taught to novices, as it is associated with a steeper learning curve and a lower complication rate for patients. However, in order to ensure that residents are adequately skilled in all situations, especially when an ultrasound machine cannot be used in an extreme emergency, they must be able to perform catheter placement by the LM technique. This study demonstrates that training in the UG technique provides the resident with certain, but insufficient skills for catheter placement by the LM technique. Residents still require a training program comprising at least 10 LM procedures to achieve optimal skills. In view of the complication and failure rates associated with these procedures, it is unclear whether or not the LM technique should still be taught to novice residents and which alternative method could be use in emergency situations when an ultrasound machine is not available.

## Key messages

• The real-time ultrasound-guided technique is the recommended procedure for central vein catheterization. However, in emergency situations an ultrasound machine may be unavailable.

• To ensure that physicians are adequately skilled in all situations, they must be able to perform catheter placement without ultrasound.

• Training in the ultrasound-guided technique provides the resident with certain, but insufficient skills for catheter placement by the landmark technique.

• A training program comprising at least 10 landmark procedures is required to achieve optimal skills.

## Abbreviations

BMI: body mass index; CVC: central venous catheter; DBP: diastolic blood pressure; GEE: generalized estimating equations procedure; LM: landmark; SAPS2: simplified acute physiologic score 2; SBP: systolic blood pressure; UG: ultrasound-guided.

## Competing interests

All authors declare that they have no competing interests.

## Authors’ contributions

JM, LG, PH, SSM and BC contributed to the acquisition and analysis of data. JM drafted the manuscript. JM, GC and MS contributed to the conception and design of the study. JM, LG, PH, SSM, BC, GC and MS were involved in the interpretation of data and critically revising the manuscript. All authors read and approved the final manuscript.

## References

[B1] Troianos CA Hartman GS Glas KE Skubas NJ Eberhardt RT Walker JD Reeves ST Councils on Intraoperative Echocardiography and Vascular Ultrasound of the American Society of Echocardiography: Guidelines for performing ultrasound guided vascular cannulation: recommendations of the American Society of Echocardiography and the Society of Cardiovascular Anesthesiologists J Am Soc Echocardiogr 2011 24 1291 1318 10.1016/j.echo.2011.09.021 22115322

[B2] Rupp SM Apfelbaum JL Blitt C Caplan RA Connis RT Domino KB Fleisher LA Grant S Mark JB Morray JP Nickinovich DG Tung A Practice guidelines for central venous access: a report by the American Society of Anesthesiologists Task Force on Central Venous Access Anesthesiology 2012 116 539 573 10.1097/ALN.0b013e31823c9569 22307320

[B3] National Institute for Clinical Excellence NICE Technology Appraisal No 49: guidance on the Use of Ultrasound Locating Devices for Placing Central Venous Catheters [http://www.nice.org.uk/nicemedia/live/11474/32461/32461.pdf]

[B4] Monnet X Lefrant JY Teboul JL Field 6. Safety practices for haemodynamic procedures (administration of vasoactive drugs, vascular and cardiac catheterization). French-speaking Society of Intensive Care. French Society of Anesthesia and Resuscitation Ann Fr Anesth Reanim 2008 27 e91 e99 10.1016/j.annfar.2008.09.012 18952404

[B5] Lamperti M Bodenham AR Pittiruti M Blaivas M Augoustides JG Elbarbary M Pirotte T Karakitsos D Ledonne J Doniger S Scoppettuolo G Feller-Kopman D Schummer W Biffi R Desruennes E Melniker LA Verghese ST International evidence-based recommendations on ultrasound-guided vascular access Intensive Care Med 2012 38 1105 1117 10.1007/s00134-012-2597-x 22614241

[B6] Feller-Kopman D Ultrasound-guided internal jugular access: a proposed standardized approach and implications for training and practice Chest 2007 132 302 309 10.1378/chest.06-2711 17625091

[B7] Denys BG Uretsky BF Reddy PS Ultrasound-assisted cannulation of the internal jugular vein. A prospective comparison to the external landmark-guided technique Circulation 1993 87 1557 1562 10.1161/01.CIR.87.5.1557 8491011

[B8] Hind D Calvert N McWilliams R Davidson A Paisley S Beverley C Thomas S Ultrasonic locating devices for central venous cannulation: meta-analysis BMJ 2003 327 361 10.1136/bmj.327.7411.361 12919984 PMC175809

[B9] Gilbert TB Seneff MG Becker RB Facilitation of internal jugular venous cannulation using an audio-guided Doppler ultrasound vascular access device: results from a prospective, dual-center, randomized, crossover clinical study Crit Care Med 1995 23 60 65 10.1097/00003246-199501000-00012 8001387

[B10] Slama M Novara A Safavian A Ossart M Safar M Fagon JY Improvement of internal jugular vein cannulation using an ultrasound-guided technique Intensive Care Med 1997 23 916 919 10.1007/s001340050432 9310813

[B11] Leidel BA Kirchhoff C Bogner V Braunstein V Biberthaler P Kanz KG Comparison of intraosseous versus central venous vascular access in adults under resuscitation in the emergency department with inaccessible peripheral veins Resuscitation 2012 83 40 45 10.1016/j.resuscitation.2011.08.017 21893125

[B12] Woo MY Frank J Lee AC Thompson C Cardinal P Yeung M Beecker J Effectiveness of a novel training program for emergency medicine residents in ultrasound-guided insertion of central venous catheters CJEM 2009 11 343 348 19594973 10.1017/s1481803500011398

[B13] Barsuk JH McGaghie WC Cohen ER O'Leary KJ Wayne DB Simulation-based mastery learning reduces complications during central venous catheter insertion in a medical intensive care unit Crit Care Med 2009 37 2697 2701 10.1097/CCM.0b013e3181a57bc1 19885989

